# Emergence of Seaweed and Seaweed-Containing Foods in the UK: Focus on Labeling, Iodine Content, Toxicity and Nutrition

**DOI:** 10.3390/foods4020240

**Published:** 2015-06-15

**Authors:** Maria Bouga, Emilie Combet

**Affiliations:** Human Nutrition, School of Medicine, College of Medical, Veterinary and life Sciences, University of Glasgow, Glasgow G31 2ER, UK; E-Mail: Maira.Bouga@glasgow.ac.uk

**Keywords:** seaweed, edible algae, iodine, market, consumer, availability, retail landscape, labelling, functional ingredient

## Abstract

Seaweed (edible algae) is not a staple food in the Western diet, despite occasional use as a traditional ingredient in coastal areas. High nutritional value, combined with the expansion of the health-food industry, has led to a resurgence of seaweed in the British diet. While seaweed could be useful in tackling dietary iodine insufficiency, consumption of some species and sources of seaweed has also been associated with risks, such as toxicity from high iodine levels, or accumulation of arsenic, heavy metals and contaminants. The current retail level of seaweed and edible algae in the UK market, either as whole foods or ingredients, was evaluated with particular focus on labelling and iodine content. Seaweed-containing products (*n* = 224) were identified. Only 22 products (10%) stated information regarding iodine content and another 40 (18%) provided information sufficient to estimate the iodine content. For these products, the median iodine content was 110 μg/g (IQR 21–503) and 585 μg per estimated serving (IQR 105–2520)*.* While calculations for iodine exposure per serving relied on assumptions, 26 products could potentially lead to an iodine intake above the (European) tolerable adult upper level of 600 μg/day. In the context of the data presented, there is scope to improve product labelling (species, source, processing, content).

## 1. Introduction

Seaweed or edible algae, a food produced on the coast of many countries, is a rich source of micronutrients. Despite high availability, low cost, and historical use in specific regions as fertiliser, animal fodder, medicine, cosmetics and even folklore, seaweed is not part of the modern Western diet [1]. Regular seaweed consumption has in some instances been associated with concerns over toxicity, ingestion of contaminants, heavy metals [2], and intake of high levels of iodine. However, seaweed is widely present in the Asian diet, and it has been found to have health benefits and possible benefit against chronic diseases, such as cardiovascular disease, cancer and diabetes, according to observational studies in South East Asia [3,4].

The concerns over toxicity and exposure to high level of iodine are mitigated by scrutiny of the seaweed species (there are more than 50 commonly eaten species) and the waters in which it was farmed or harvested. For example, the iodine content of seaweed varies from 16 μg/g in some Nori species (*Porphyra tenera*) to 8165 μg/g in Icelandic Fingered tangle [5]. Meanwhile, inorganic arsenic concentration is found to be low in kelp species, one of the most common edible seaweed category, and within the Tolerable Daily Intake level of 2 μg/kg body weight, set by the World Health Organisation (WHO) [6].

Overall, there is lack of data regarding seaweed availability and consumption in the British diet, with limited consumer knowledge on the product(s) [7]. There is potential for seaweed to act as a functional food and ingredient [8], relative to its role as a rich source of iodine [9], its antimicrobial properties against mainly Gram negative microorganisms [10] and satiating properties when included in food products such as beverages, breakfast bars and pizza [11,12], and use in low sodium salts. The role of seaweed as a rich source of iodine is particularly relevant in the UK, where recent surveys have highlighted insufficiency in different groups of the population [13,14,15,16], with iodine being an essential component of thyroid hormones, which are essential for neurodevelopment in utero and after birth [17,18].

The main dietary sources of iodine in the UK are dairy products, mainly milk, and seafood. However, fish consumption is low in the British population and milk consumption is decreasing [19]. In 2007, 130 countries and almost 70% of the world population were using iodised salt [20], according to WHO recommendations for universal salt iodisation [21]. However, in the UK, there is no prophylaxis, the availability of iodine rich salt in the market is very low [22], compounded by low awareness about iodine importance in pregnancy and poor knowledge of iodine-rich foods [23]. Recent coverage in the UK media also advocated for a seaweed-based diet, in response to low iodine intake in the population, which is potentially harmful, since it may lead to over-exposure to the nutrient [24].

Retail availability and intake or iodine-rich foods are essential for individuals to meet their daily iodine requirements (140 μg, equivalent to two portions of fish per week, and dairy to the equivalent of one glass (drinks, in cereals), plus a cheese serving per day). Seaweed, consumed as a whole product or incorporated in other foods has potential as a functional ingredient to increase iodine availability in the food chain [25], as long as micronutrient content of the seaweed is carefully evaluated to avoid unintentional exposure to (too) high doses of iodine.

The aims of this study are to identify the availability of seaweed and seaweed-containing products in the UK market, and to collate data on iodine content of the foods and its reporting on product labels.

## 2. Methods

### 2.1. Data Collection

A product survey took place in June 2014 to identify UK retailed products that contained seaweed. The product lists of all UK grocery retailers were searched, with the exception of those that did not have an online shop. Twenty nine different retailers, which all have an online shop, were included in the survey. Product selections available for purchase were searched with the keywords “seaweed”, “sea vegetable”, “Ascophyllum”, “Laminaria”, “lava bread”, “miso soup”, “Arame”, “Kombu”, “kelp”, “algal” and “Dulse”.

### 2.2. Identification of Products’ Iodine Information

All products’ packaging was checked for information relative to their iodine content. All products’ labels which detailed iodine content were recorded in the database. If a product label detailed a range of iodine content, the average content was recorded. Some products (*n* = 8, 4%) had only qualitative description of their iodine content (*i.e.*, “rich in iodine”, “a very good source of iodine”), in which case no iodine content was recorded. For products labels detailing the type of seaweed and percentage of seaweed content, assumptions were made to estimate their iodine content, based on previous data published by Teas, Pino, Critchley and Braverman [5], Romaris-Hortas, *et al.* [26], Lee, *et al.* [27], van Netten, *et al.* [28], MacArtain, Gill, Brooks, Campbell and Rowland [9], Gall Erwan, *et al.* [29], Nagataki [30], Watanabe, *et al.* [31] and Aquaron, *et al.* [32], using average values when more than one study had iodine information for a seaweed type.

### 2.3. Other Data Collected

Price was recorded. When more than one retailer sold the product, the average price was used.

Serving size was recorded. If a product was available in more than one serving size, the largest serving size was recorded. Portion sizes were determined following recommendations on the package. If not serving size suggestions were made, portion sizes were estimated according to the food category, using standard portion sizes outlined in the Windiets Version 2005 dietary analysis software.

### 2.4. Data Analysis

Descriptive statistics were carried out with the statistical software SPSS Version 21.0 (IBM Corp., Armonk, NY, USA). Normality was tested with the Kolmogorov-Smirnov normality test.

## 3. Results

### 3.1. Seaweed Products Availability

The seaweed products were identified in 29 different retailers, covering 82.2% of the UK grocery market share [33], from which only 17 were selling on the high street. A total of 226 single products were identified. Two were excluded from the analysis as they contained the “seaweed” keyword but not any seaweed (the labels included “spring cabbage”, which is not a seaweed type). All surveyed retailers had an online presence. Seven retailers were considered to be large supermarkets in the UK, selling 30% (*n* = 66) of the product range, with the other 22 specialist shops selling 70% (*n* = 158) of the product range. All products were sold either in a supermarket or in a specialist shop. Most products originated from the UK (63%, *n* = 142), Japan (9%, *n* = 18), China (2%, *n* = 4), New Zealand, (0.4%, *n* = 1), Thailand (0.4%, *n* = 1) and Switzerland (0.4%, *n* = 1); other products (25%, *n* = 56) had no origin labelled on their packaging.

Sixty five percent (*n* = 146) of the seaweed and seaweed-containing products were sold solely online, while the rest (*n* = 78, 35%) could be found both online and on the high street. The median price of the products was £4.00 (interquartile range (IQR) 3.00–6.80), with the less expensive product being sold £0.69 and the most expensive £55.00. Median packaging size of the products, measured in grams, was 134 g (IQR 50–345).

The 224 products belonged to 10 different product categories including bread and confectionery, condiments, drinks, noodles and pasta, salads, seaweed, snacks, soup, supplements and sushi (Table 1). Only one of them did not belong to any of these categories. This product was a “gelatine alternative” described as “other” in Table 1.

**Table 1:** Number of products containing seaweed in each set product category.

Product Category	*n* of Products (*n* = 224)	% of Products	% Retailed in Supermarkets	Products Examples
Bread and Confectionery	42	19	0	Bread, cake, pizza base, biscuits, shortbread
Condiments	43	19	7	Seaweed flakes, salad booster, salt
Drinks	5	2	20	Gin, whisky, super shake, smoothie
Noodles and Pasta	9	4	33	Sea spaghetti, kelp noodles
Salads	7	3	0	Seaweed salad, sea salad
Seaweed	52	23	14	Whole seaweed, seaweed sheets
Snacks	8	4	75	Crackers, rice crackers, oatcakes
Soup	15	7	100	Miso soup
Supplements	11	5	0	Tablets
Sushi	31	14	100	Sushi platters
Other	1	0	0	Gelling Agent

### 3.2. Types of Seaweed

The seaweed species contained in each product were identified, with common and scientific name presented in Table 2, alongside the estimated iodine content of each species. Thirty five products (16%) did not have any visible information regarding the seaweed type contained and 12 (6%) did not specify the seaweed type. From the remaining 177 food products that had information about the contained seaweed type, 35% (*n* = 62, belonging to 6 categories: Bread and confectionery, condiments, drinks, noodles and pasta, snacks and supplements) used a trademarked seaweed ingredient (Seagreens^®^ *Ascophyllum nodosum* containing 700 μg iodine per g of dried seaweed, *Fucus vesiculosus* 522 μg per g of dried seaweed or *Pelvetia canaliculata* containing 243 μg per g of dried seaweed). Nori was the next more commonly found seaweed type, used in 24 products (14%), followed by Wakame (*n* = 21, 12%), Kelp (*n* = 20, 11%) and Dulse (*n* = 14, 8%). Table 2 presents in detail the emergence frequency of all seaweed types as well as the estimated iodine content of each seaweed type in known cases.

### 3.3. Iodine Content of Seaweed Products

**Figure 1:**
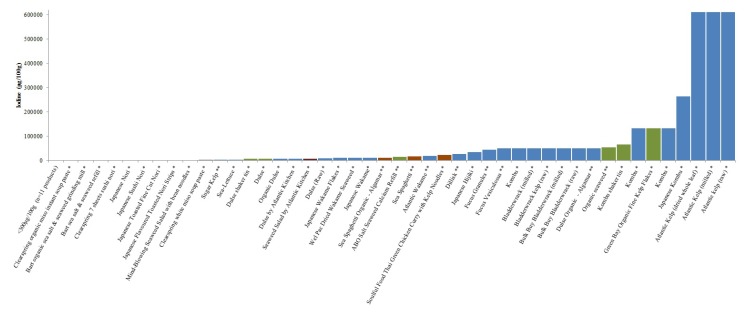

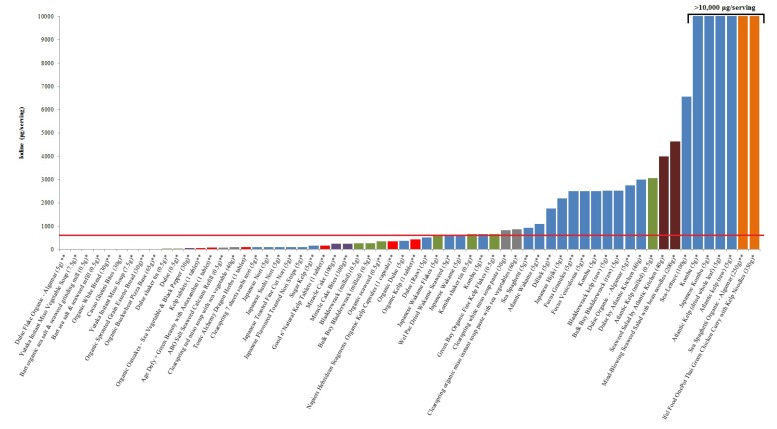
(**A**) Iodine content (μg/100 g) of identified seaweed products with known iodine concentration; (**B**) Iodine content (μg/serving) of identified seaweed products with known iodine concentration. Bar colour indicates the product categories: ● seaweed; ● salad; ● condiments; ● soup; ● noodles and pasta; ● supplements; ● bread and confectionary. Red line indicates the European tolerable upper level for iodine (600 μg/day) [37]. Products with one asterix (*) have estimated iodine content and with two asterix (**) are products for which iodine content was provided. Estimated serving size is indicated in bracket4. Discussion

Only 22 products (10%) clearly stated quantitative information on iodine content and another 40 products (18%) provided information that enabled estimation of the iodine content (Figure 1). In these products, median iodine content of the food products was 110 μg/g (IQR 21–503) and 585 μg per estimated serving (IQR 105–2520). Median iodine content of supplements was 127 μg per capsule or tablet (IQR 65–368). The majority of the identified seaweed products in the UK grocery market (63%, *n* = 141) did not have any labelling information relative to their iodine content and did not provide any information from which iodine content could be retrieved or estimated (*i.e.*, unknown seaweed type, unknown seaweed content in the product composition, unknown seaweed iodine content). The range of seaweed content in the products was 0.1%–100%. In addition, packaging information on 21 products (9%) was not available online and information on iodine could not be retrieved. While calculations for iodine exposure per serving relied on assumptions, 26 products could potentially lead to an iodine intake above the European tolerable upper intake level (TUL) of 600 μg/day [34] and 19 above the adult upper level of tolerance of 1100 μg/day set by the Institute of Medicine [35].

**Table 2:** Common and scientific names of seaweed types, their emergence, iodine content, and derived iodine content in products.

Common Name	Species	Fresh	Dried	*n* of Products Containing that Species		*n* of Products with Derivable iodine Content
		Average iodine content (μg/g) *		*n*	% ^†^	*n*
Fingered tangle	*Laminaria digitata*	700 [9]	6118 [5,9,29]	5	3	3
Kelp	Median value		1327	20	11	2
	*Laminaria longicruis*		1304 [5]			
Bull Kelp	*Nereocytis leutkaena*		407 [28]			
Split kelp	*Laminaria setchelli*		1070 [28]			
Sugar kelp	*Laminaria Saccharina*		238 [28]			
Winged kelp	*Alaria marginata*		151 [28]			
Giant kelp	*Macrocystis integrifolia*		240 [28]			
Kombu	Median value		2650	9	5	7
	*Laminaria japonica*		2380 [5,27,28,30]			
	*Laminaria ochroleuca*		6138 [26]			
Hijiki	*Hizikia fusiforme*		436 [5,27,28]	2	1	1
Wrack	Median value	182	725			
Egg wrack.	*Ascophyllum nodosum*	182 [9]	725 [5]	62	32	0
Bladderwrack	*Fucus vesiculosus*		504 [5,28]	9	5	6
Wakame	Median value	39	172	21	12	11
	*Undaria pinnatifida*	39 [9]	189 [5,9,27,28,30]			
	*Undaria pinnatifida*		139 [27,28]			
	*Alaria esculenta*					
Sea spaghetti	*Himanthalia elongata*	107 [9]	117 [5,9,26]	3	2	1
Dulse	*Palmaria palmata*	102 [9]	75 [5,9,26,27]	14	8	7
Sea lettuce	Median value	16	90	11	6	2
	*Ulva lactuca*	16 [9]	114 [5,9]			
	*Ulva rigida*		66 [26]			
Nori	Median value		21	24	14	7
	*Porphyra purpurea*, *Porphyra tenera*, *Porphyra yezoensis*		11 [27,28]			
	*Porphyra tenera*		34 [5,27,28,31]			
Irish moss	*Chondrus crispus*	61 [9]	238 [9]	1	1	0
Chlorella	*Chlorella sp.*			4	2	0
Gracilaria	*Gracilaria verrucosa*			3	2	0
Laver	Median value	15	117	5	3	0
	*Ulva pertusa*	16 [9]	163 [9]			
	*Porphyra umbilicalis*	13 [9]	80 [9,26]			
Pelvetia	*Pelvetia canaliculata*		243 [36]	5	3	2
Sea belt	*Laminaria saccharina*, *Saccharina latissima*			5	3	0
Other Shony Agar Sea fern Japanese moss Grapestone				14	6	1

* Information retrieved from Teas, Pino, Critchley and Braverman [5], Romaris-Hortas, Garcia-Sartal, del Carmen Barciela-Alonso, Dominguez-Gonzalez, Moreda-Pineiro and Bermejo-Barrera [26], Lee, Lewis, Buss, Holcombe and Lawrance [27], van Netten, Hoption Cann, Morley and van Netten [28], MacArtain, Gill, Brooks, Campbell and Rowland [9], Gall Erwan, Küpper Frithjof and Kloareg [29], Nagataki [30], Watanabe, Takenaka, Katsura, Masumder, Abe, Tamura and Nakano [31] and Aquaron, Delange, Marchal, Lognone and Ninane [32]. Iodine content of each type calculated as median when more than one value was present in these studies. Missing data are presented as blank cells. ^†^ Percentage of products containing the specific seaweed type, based on products that have labelled seaweed type (*n* = 177).

## 4. Discussion

### 4.1. Study Findings and Seaweed Safety

Seaweed was never staple food in the Western world, despite traditional uses, such as medicine, cosmetic, and folklore [1]. However, in recent years, the potential of seaweed use as a functional ingredient has arisen [8] potentially because of the increasing influence of Asian cuisine and the apparent health benefits associated with its consumption [10]. In Japan, seaweed is a component of the habitual diet (as high as 5.3 g/day) [38] and has been linked with health benefits, including reduced incidence of cancers, hyperlipidaemia, coronary heart disease (CHD) and metabolic syndrome, digestive track and bone health and antiviral properties [3].

In this study, we have shown that seaweed is available both as a whole product and an ingredient, in a diversified range of food products, even if it still remains a “specialist shop” product (with only 30% of the identified products being sold in large supermarkets). Even though seaweed is not integrated in the Western diets, this study found that the vast majority of the seaweed products had UK origin. According to the origination rules, the origin of a food product is the last country in which a food is substantially changed, so if a product is only packaged in a country it cannot be considered to originate from there. However many of these products did not state a country of harvest and a country of packaging and when UK was in the label they were considered as UK originated.

We have previously shown that 0.5 g seaweed intake, equivalent to 356 μg iodine per day, could increase the iodine status of women [7]. However, the iodine content in seaweed-containing food was reported in only 10% of the products, and estimated based on compositional information in another 18%, leaving 72% with no derivable information. Considering the large variability in the iodine content of seaweed species [26,30], and the TUL for iodine (600 μg/day) [37], this absence of information is a potential safety issue.

The EFSA upper intake level of 600 μg/day is derived from studies with no observed clinical adverse effect, with humans exposed to up to 1800 μg iodine/day. This no-observed adverse effect level, corrected for an uncertainty factor of 3 (relative to the number of studies available to extrapolate the TUL, their duration and sample sizes) gave a TUL of 600 μg/day. The EFSA concluded that consumption of high amount of edible algae may be harmful, in respect to the variable iodine content. This is potentially also even more relevant considering that the TUL is designed for the general population, rather than populations with iodine deficiency, who have increased sensitivity to iodine [39].

Exposure to excess iodine can lead to formation of a goiter, hypothyroidism or hyperthyroidism and iodism in case of chronic exposure. Large (excessive) iodine intake can inhibit the formation of thyroid hormones and increase plasma TSH, a phenomenon known as the Wolff-Chaikoff effect, which is transient. In vulnerable groups with autoimmune thyroid disease, excessive iodine intake can also lead to thyroiditis, sensitivity reactions, and papillary thyroid cancer [10,40,41]. There was however no direct association between seaweed consumption and thyroid cancer when healthy Japanese women were studied [42]. The present study identified a range of products (*n* = 26) which could provide higher iodine intake than the TUL of 600 μg/ day [37], raising the concern of the safety of these products consumption in a regular basis.

However, not all seaweed species have very high iodine content. Compositional information on seaweed products is important in order to estimate their iodine content. In cases of very high iodine intake from soya milk fermented with iodine in Australia in 2009, conditions ranging from florid thyrotoxicosis to subclinical hypothyroidism were observed. The urinary iodine concentrations of these patients were also much higher than the reference range of 200 μg/L. After ceasing the consumption of this soy milk, thyroid function normalised again [41].

### 4.2. Functional Potential of Seaweed

Seaweed is included in products composition as a salt substitute to enhance taste, food matrix or to enrich the product with natural bioactive compounds [10]. In this way, seaweed can be considered as a product that has the potential to benefit the food industry and the health of the population through its growing use. Seaweed products or seaweed isolated ingredients fall within the novel foods set by the European Union and should always obey to the European Regulation on Novel Foods (258/1997). There are currently five unauthorized claims in the European register on nutrition and health claims [43], pertaining to the use of seaweed for the management of blood glucose and insulin levels, body detoxification and appetite and hunger control [43].

Seaweed is a good iodine source and its contribution to the daily iodine intake of the UK population should be further explored. Accurate laboratory analysis is needed to define seaweed products’ iodine content. Estimating iodine content of seaweed products can lead to under or overestimations, as the exact iodine content of each seaweed might differ depending on the processing or the exact species (e.g., kelp is labelled as a common name in many food products but can refer to many different species [5], which might differ significantly in their iodine content). There are six authorised claims for iodine in the European register on nutrition and health claims [43]. The six claims highlight that iodine contributes to normal cognitive function, normal energy-yielding metabolism, normal functioning of the nervous system, the maintenance of normal skin, the normal growth of children, the normal production of thyroid hormones and normal thyroid function. There are three unauthorized claims relative to iodine for maintenance of vision, hair and skin.

Seaweed is now available on the UK market for use both as a whole food and as an ingredient in many different food products. It is always labelled in the package but without reporting usually the iodine concentration of the products or the exact amount of seaweed used in the recipe or formulation. Iodine should be considered for analysis, as very high concentrations might cause toxicity and adverse effects but safe levels might be able to help reduce the iodine insufficiency in the UK population. There should be careful consideration on iodine levels in products using seaweed as a functional food. Inclusion of nutrient-dense ingredients in specific food formulation will also drive a need to revise food composition table, which may considerably skew the iodine content for some food groups and render dietary assessment difficult.

### 4.3. Limitations and Future Work

The cross-sectional design of the present study does not allow us to draw conclusions on the impact of these products on consumers and provides results only for the selected time-frame, in a young, very dynamic market. The identification of the seaweed products was carried out online, leaving out products sold in stores with no online presence. Our survey, however, covered over 82% or the retail landscape, while omitting restaurants, catering outlets and local Asian shops that sell seaweed products.

This survey did not take in consideration contaminants and heavy metals, and there is scope in assessing these. Iodine was only labelled in a minority of the food products with limited provision of the necessary information to accurately calculate iodine content in others. At the moment, there are no rules regarding iodine and/or seaweed labelling in food products, making it difficult to retrieve information about the contribution of products containing iodine and seaweed on the daily iodine intake, as well as estimating their suitability regarding toxicity. This lack of information and the impact of processing on seaweed content limits our analysis and also highlights the need for the products to display iodine content. Cooking loss of the water-soluble iodine is another fact difficult to take into account, as it varies significantly between the cooking methods [44,45]. Iodine bioavailability is also dependent on the food matrix as was previously demonstrated *in vitro* [25,26] meaning that high iodine concentration in food may not translate to high level absorbed.

We also estimated typical serving sizes, and relied on the published literature to estimate the iodine content of seaweed, which is subject to great seasonal and analytical variability. As such, the estimations of iodine exposure are approximate.

## 5. Conclusions

The present study identified a wide range of seaweed containing products sold in the UK grocery market. The lack of information regarding the seaweed type used, its source and iodine content makes it difficult to formulate safe conclusions regarding the safety and suitability of these products and is a potential issue for high iodine exposure, especially for consumers who are pregnant. Further information on the source of seaweed (and derived information on water quality) and information on how it was processed will also enhance the ability to assess potential exposure to contaminants and toxic compounds. Owing to the nutrient density and the potential use of seaweed as a functional ingredient, this information would enhance its safe use and magnify the potential accompanied health benefits of edible algae. There is additional scope to study consumer purchase behaviour in relation to seaweed products, in terms of demographics and drivers for purchase.
